# The New Loves Gain the Day

**Published:** 1891-01-17

**Authors:** 


### PASSING TOPICS.

### THE NEW LOVES GAIN THE DAY.

Neither Lord Derby's salutary warning, upon which we commented last week, the unstinted approval of the several organs of public opinion which have dealt with it, our own modest paraphrase of the counsel offered, its own inherent reasonableness—nor all these combined—have availed to save the established institutions from the penalties of kinsmanship, and the subject is so important to our great unendowed charities that we make no apology for returning to it. Forgotten, because their work is always in evidence; overlooked, because they live under our eyes; and neglected, because they are so indispensable that their continued existence is taken for granted, many of our most useful organisations languish for want of sustenance, and are threatened with veritable starvation.

Upon all hands, we learn that our hospitals are in distress. Christmas and the New Year—those times sacred to the exercise of charity—have come and gone, and the empty coffers remain empty still. One well-known, and as it is generally believed, well-supported institution, with liabilities of more than £1,000 per month, publicly advertises the fact that the total of the benefactions received by it during the three weeks ending the 7th inst., amount to £6 and some odd shillings, and another equally deserving reports that its receipts are less than one-third of the average. At the same time "General" Booth can boast a subscription list approaching £100,000 collected and promised within a few weeks; and Mr. Balfour asks to be excused from individual acknowledgment of the gifts showered upon him, "because the rapid and generous response renders it quite impossible for me to deal personally with the mass of correspondence which would be entailed."

We do not begrudge either the one or the other his good fortune, but we cannot pretend any superabundant admiration of a generosity exercised at the expense of our established charities. It will be remembered by some of our older readers that in the one-time venerated, but now despised, history of "Sandford and Merton," Mr. Barlow had no difficulty about exposing the fictitious character of the benevolence illustrated by Tommy's gift to the beggar of loaves taken from Mr. Barlow's own pantry, and although it might be thought we were going too far if we attempted to assert for an institution a right of property in its subscribers' donations, we are well within the mark when we say that, so long as the appeal is to our sense of reason and of right, as much as to our sense of pity, there can be no withdrawal of necessary help without the perpetration of a wrong.

Of course, people are always to be found of temperament sanguine enough to enable them to take all good things for granted, and this is pre-eminently noticeable when their own lines have fallen in pleasant places. We remember to have heard a distinguished physician, recently deceased, remark that he did not admit an increase in the ravages of a particular class of diseases, because he was not prepared to believe that it was permitted to any evil thing to increase. This was optimism indeed, beside which the comfortable proposition that partial evil is universal good sinks into insignificance; and, in a similar spirit of confidence, not, as it seems to us, better justified by practical experience and the record of actual facts, a medical contemporary complacently quotes figures which it considers are sufficient to show that "charity is not failing."

Nothing can be more fallacious than to draw a conclusion of this sort from general aggregations of benefactions. During the last year one or two metropolitan hospitals have secured windfalls, and the total of receipts is respectable enough in the abstract, but, on the whole, we are inclined to echo the same journal's reflection that, as compared with the wealth of a country which pays income-tax on five hundred millions a year, "these sums are a mere drop in the bucket."

Agricola.

